# Relationship between Insulin Secretion and Arterial Stiffness in Essential Hypertension

**DOI:** 10.1155/2021/5015797

**Published:** 2021-12-24

**Authors:** Yancui Sun, Yanqiu Zhu, Lu Zhang, Yan Lu, Yan Liu, Ying Zhang, Wei Song, Yinong Jiang, Yunpeng Cheng

**Affiliations:** ^1^Department of Cardiology, The First Affiliated Hospital of Dalian Medical University, Dalian, Liaoning, China; ^2^Department of Ultrasonography, The Affiliated Hospital of Dalian University, Dalian, Liaoning, China

## Abstract

The study aims to explore the relationship between plasma insulin secretion and arterial stiffness in nondiabetic essential hypertensive patients. A total of 730 nondiabetic essential hypertensive patients registered between January 2016 and October 2020 were enrolled. A two-hour oral glucose tolerance test (OGTT) was performed to detect the levels of C-peptide and blood glucose at 0 hours and 2 hours, as well as the difference between C-peptide (Δ C-peptide) and blood glucose (Δ blood glucose) over the same period. Patients were divided into two groups: the normal glucose tolerance (NGT) group (*n* = 322) and the impaired glucose tolerance (IGT) group (*n* = 408). A multiple linear regression analysis was used to evaluate the association between brachial-ankle pulse wave velocity (baPWV) and the other factors. 0 h C-peptide, 2 h C-peptide, and Δ C-peptide were found to be higher in the IGT group. baPWV was positively linear correlated with 2 h C-peptide (*r* = 0.086, *p*=0.020) and Δ C-peptide (*r* = 0.115, *p*=0.002). baPWV remained independently associated with 0 h C-peptide, 2 h C-peptide, and Δ C-peptide, after adjusting by age, gender, smoking, body mass index (BMI), high-density lipoprotein (HDL), cholesterol, systolic blood pressure (SBP), and triglycerides (TG). Our data shows that higher endogenous insulin secretion might play an important role in the progression of arterial stiffness in nondiabetic essential hypertensive patients.

## 1. Introduction

Hypertension is a leading risk factor of cardiovascular diseases. Many studies have revealed that hypertension, in the absence of obesity or diabetes mellitus, is associated with insulin resistance [1–3]. When insulin resistance appears, insulin levels are elevated, and the resulting increase can be reflected by the detection of insulin content. C-peptide is cosecreted with insulin on an equimolar basis; this is more stable, has fewer influencing factors, and can better reflect the secretion ability of overall and residual islet *ß*-cell [4–6]. In addition, C-peptide may also be a predictive marker of the severity of cardiovascular disease [7] and mortality [8]; the research suggests that C-peptide is a bioactive peptide with other potential physiological functions. Meanwhile, the detection of C-peptide is simple and convenient.

Arterial stiffness is accompanied by an increase of pulse wave velocity, which is an important predictor of cardiovascular disease [9–12]. Several factors are believed to be responsible for arterial stiffness, including age, hypertension, and hyperglycemia. Studies have found that insulin resistance is significantly associated with arterial stiffness [13, 14]. Euglycemic hyperinsulinemic clamp studies have revealed a relationship between insulin resistance and arterial stiffness in both young, healthy women and diabetic adults [15–17]. A few studies examining the existence of this relationship were conducted for individuals without diabetes. Additionally, studies involving the association between insulin secretion and arterial stiffness are limited.

Therefore, this study aims to determine the association between insulin secretion (assessed by C-peptide) and arterial stiffness (assessed by brachial-ankle pulse wave velocity (baPWV)) in the Chinese essential hypertensive population without diabetes.

## 2. Methods

### 2.1. Study Subjects

All subjects in this study were examined within the period of January 2016 to October 2020 at the First Affiliated Hospital of Dalian Medical University. The inclusion criteria are as follows: (1) hypertensive patients classified according to the 2018 European Society of Cardiology and the European Society of Hypertension (ESC/ESH) guidelines office [18], with systolic blood pressure (SBP) at a value ≥ 140 mmHg or diastolic blood pressure (DBP) at a value ≥ 90 mmHg, using antihypertensive drugs, and (2) ≥18 years old. The exclusion criteria are as follows: (1) diagnosis of secondary hypertension (*n* = 106), (2) diagnosis of diabetes mellitus (*n* = 493), (3) severe arrhythmia (*n* = 34), (4) moderate to severe kidney dysfunction (estimated glomerular filtration rate (eGFR) ≤60 ml min^−1^ 1.73 m^2^) (*n* = 122), (5) left ventricular systolic dysfunction (LVEF <50%) (*n* = 58), (6) severe valvular heart disease (*n* = 26), (7) peripheral vascular diseases (PAD) (*n* = 62), (8) myocardial infarction or unstable angina pectoris (*n* = 156), and (9) those subjects missing dates of baPWV or C-peptide (*n* = 213). Finally, the present study contained a total of 730 subjects. The research was conducted in accordance with the Declaration of Helsinki and was approved by the institutional review board of the First Affiliated Hospital of Dalian Medical University. The informed consent provision was waived, and all procedures listed here were carried out in compliance with the approved guidelines.

### 2.2. Clinical Measurements and Definition of Explanatory Variables

Demographic and clinical characteristics include age, gender, height, weight, whether the subject is a current smoker, cardiovascular disease, use of oral antihypertensive drugs, and use of statins. Participants were deemed current smokers if they reported that they were currently smoking or had registered smoking at least 100 cigarettes during their lifetime [19, 20]. Body mass index (BMI) was calculated according to the following formula: weight (kg)/height (m)^2^. A sample of fasting blood from the brachial vein was collected, and the biochemical parameters included total cholesterol (TC), triglycerides (TG), low-density lipoprotein (LDL) cholesterol, and high-density lipoprotein (HDL) cholesterol. The sample collections were performed at the First Affiliated Hospital of Dalian Medical University using the standard protocols. eGFR was calculated according to the equation suggested by the Chronic Kidney Disease Epidemiology (CKD-EPI) Collaboration [21]. PAD was defined as ankle-brachial index (ABI) < 0.9, stent implantation, or according to past medical records.

### 2.3. Oral Glucose Tolerance Test (OGTT)

Blood samples for glucose and C-peptide were obtained at 0 hours and 2 hours after a 75-g oral glucose load. Serum C-peptide levels were measured by chemiluminescence immunoassay, while serum glucose concentrations were assayed using a glucose oxidase assay. Hemoglobin A1c (HbA1c) analysis was performed on whole blood using high-performance liquid chromatography. The diagnosis of diabetes and prediabetes was defined based on the World Health Organization's 1999 criteria after the OGTT [22]. Participants were divided into two groups: the normal glucose tolerance (NGT) group and the impaired glucose tolerance (IGT) group. NGT was diagnosed if the fasting plasma glucose level was <6.1 mmol/L and the 2 h value was <7.8 mmol/L. IGT was diagnosed if the fasting plasma glucose level was <7.0 mmol/L but the 2 h value was 7.8–11.1 mmol/L, or if fasting glucose was 6.1–7.0 mmol/L but the 2 h glucose value was <7.8 mmol/L.

The plasma glucose difference (Δ plasma glucose) was defined as 2 h plasma glucose – 0 h plasma glucose, whereas the C-peptide difference (Δ C-peptide) was defined as 2 h C-peptide – 0 h C-peptide.

### 2.4. Ambulatory Blood Pressure

Blood pressure was measured on an ambulatory basis using an automatic 24-hour monitor (model 90207; Spacelabs, Issaquah, WA). Measurements were taken every 30 minutes during the daytime period (0700–2300 h) and every hour during the night period (2300–0700 h). These measurements were averaged to produce the mean 24 h systolic blood pressure (SBP), diastolic blood pressure (DBP), and heart rate (HR).

### 2.5. Measurement of baPWV

baPWV measurements recorded in the supine position were obtained using a volume-plethysmography device (VP-1000, OMRON, Dalian, China), which measures both brachial and posterior tibial artery pressure waveforms using an oscillometric method with cuffs placed around the participant's arms and ankles. baPWV was calculated automatically via time-phase analysis, and the distance between the upper arm and ankle was estimated based on the participant's height. baPWV was obtained from right and left measurements, and the higher of the two readings was used for analysis [23].

### 2.6. Statistical Analysis

All statistical analyses were conducted using SPSS version 26.0. Normally distributed continuous variables were recorded as mean ± standard deviation. Nonnormal distribution parameters were transformed or presented as the median (25th –75th percentile). Categorical data were presented as number and percentages (*n*, %). Differences between groups were compared by using Student's *t*-test, the Chi-squared test, and Mann–Whitney's *U* test. Spearman correlation or Kendall correlation analysis was used to explore the association between baPWV and other factors, while multiple linear regression analysis was used to evaluate this association. All statistical analyses were two-sided, and a *P* value of less than 0.05 was considered statistically significant.

## 3. Results

### 3.1. Baseline Characteristics of the Participants

During the study period, 730 subjects had sufficient data to evaluate the baPWV and C-peptide. The baseline characteristics of participants are shown in Table 1. The mean levels of BMI, 0 h plasma glucose (0 h PG), 2 h plasma glucose (2 h PG), Δ plasma glucose, 0 h C-peptide, 2 h C-peptide, Δ C-peptide, HbA1c, and TG in the IGT group were found to be significantly higher than those in the NGT group (all *P* < 0.05). Meanwhile, the mean level of HDL-C was lower in the IGT group (*P* < 0.001). Other factors include age, female, current smoker, eGFR, TC, LDL-cholesterol, HR, SBP, DBP, baPWV, stable angina, use of antihypertension drugs, and use of statin were not statistically significant in two groups.

### 3.2. Univariate Correlation with baPWV

As shown in Table 2, correlation analysis indicated that baPWV was positively correlated with age (*r* = 0.407, *P* < 0.001), 2 h C-peptide (*r* = 0.086, *P*=0.020), Δ C-peptide (*r* = 0.115, *P*=0.002), HDL-C (*r* = 0.158, *P* < 0.001), HbA1c (*r* = 0.077, *P*=0.037), SBP (*r* = 0.211, *P* < 0.001), stable angina (*r* = 0.081, *P*=0.029), calcium channel blocker (CCB) (*r* = 0.160, *P* < 0.001), diuretics (*r* = 0.167, *P* < 0.001), and statin (*r* = 0.178, *P* < 0.001). The other factors, namely, female (*r* = −0.181, *P* < 0.001), current smoking (*r* = −0.151, *P* < 0.001), BMI (*r* = −0.235, *P* < 0.001), eGFR (*r* = −0.125, *P*=0.001), and TG (*r* = −0.100, *P*=0.007) were found to be negatively correlated with baPWV.

### 3.3. Association between baPWV and the C-Peptide Correlation Index

The results of the multiple linear regression models used to determine the degree to which each of the different covariates impacted baPWV are presented in Table 3 as *ß* and 95% confidence intervals (CIs). Model 1 was not adjusted, model 2 was adjusted for age and sex, and model 3 was adjusted for age, sex, current smoking, BMI, HDL-C, mean 24h SBP, and TG. After controlling for confounding factors, it was found that 0 h C-peptide (*β* = 0.091, *P*=0.012), 2 h C-peptide (*β* = 0.089, *P*=0.006), and Δ C-peptide (*β* = 0.078, *P*=0.014) were positively associated with baPWV.

## 4. Discussion

Hypertension is associated with insulin resistance (IR), metabolic syndrome (MS), and arterial stiffness [24]. Some studies show there is a correlation between insulin resistance and arterial stiffness [15–17]; however, there is a lack of research on the relationship between insulin secretion and arterial stiffness. The research paper aims to explore the relationship between insulin secretion and arterial stiffness.

Although C-peptide is not a direct measure of *ß* cell mass, its plasma levels provide a fairly accurate estimate of endogenous insulin secretion. Some studies show that assessing the *ß*-cell function as a measurement of C-peptide after stimulation may be advantageous [25]. 2 h C-peptide represents the insulin content after 2 hours of glucose tolerance, while Δ C-peptide reflects the ability of pancreatic islet *ß* cells to secrete insulin after glucose tolerance. The main finding of this study is that 0 h C-peptide (*β* = 0.091, *P*=0.012), 2 h C-peptide (*β* = 0.089, *P*=0.006), and Δ C-peptide (*β* = 0.078, *P*=0.014) are associated with baPWV, and this association appeared to be independent of glucose tolerance status in hypertensive nondiabetic subjects. Furthermore, the association remained positive after adjusting for age, sex, BMI, and other confounding factors. Higher Δ C-peptide may represent higher endogenous insulin secretion and may be a compensatory factor of insulin resistance due to higher baPWV, which implies that higher Δ C-peptide plays an important role in the progress of arterial stiffness.

Previous studies have shown that age, gender, and BMI are associated with increased arterial stiffness [26, 27]. The present study confirms the association between age, gender, BMI, and arterial stiffness in this population. The study also found, through the multiple regression models, that the relation between insulin secretion and arterial stiffness was independent of age, gender, and BMI. These results suggest that the relationship between age, gender, or BMI and arterial stiffness may be mediated in part through the increase of insulin secretion.

The mechanism underlying the relationship between insulin secretion and arterial stiffness is unknown, and this cross-sectional study cannot identify the causative factor. The hyperinsulinemic response by the *ß* cells is multifactorial, including adaptive changes in *ß* cell mass and function. Related literature has shown that the increase of insulin secretion is reflected by the mass of islet cells rather than the secretion capacity of pancreatic *ß* cells [28–30]. The present study finds that, under a long-term high-pressure environment, *ß* cells initially mount a compensatory response to ramp up *ß* cell functional capacity and insulin secretion to meet the elevated pressure demand. Other studies have found that insulin resistance leads to arterial stiffness [13, 14, 31]. The outcome of the abovementioned mechanisms is increased insulin secretion. Abnormal secretion of insulin can enhance the activity of the renin-angiotensin-aldosterone system (RAAS) and the sympathetic nervous system. The results of these changes involve the increase in the secretion of angiotensin, aldosterone, catecholamine, and endothelin 1, as well as the promotion of the contraction and remodeling of blood vessels, leading to arterial stiffness [32]. In addition, excessive RAAS activation increases the expression of distal renal tubular sodium channels, enhances the activity of vascular endothelial sodium channels, and promotes fibrosis, remodeling, diastolic dysfunction of arterial and salt sensitivity in blood pressure regulation. Finally, these changes aggravate blood pressure and the progression of arterial stiffness [33].

This study did not identify an increase in arterial stiffness among individuals with IGT. This finding was consistent with previous studies that had failed to identify a significant increase in central arterial stiffness measurements among those with IGT compared with those with NGT [31]. A previous study found that hyperglycemia is associated with significant arterial stiffening and that postchallenge glucose and the homeostasis model assessment of insulin resistance (HOMA-IR) index are the most powerful metabolic predictors of arterial stiffness [34]. This research, however, finds no association between arterial stiffness and glucose measurements including 0 h PG, 2 h PG, and Δ plasma glucose level. The mechanism underlying the relationship is unknown, and this cross-sectional study cannot identify the causative factor; however, the duration of diabetes and the level of blood glucose may be explored in future studies.

It is important to note several limitations in this study design. First, the cross-sectional design restricts the causal relationship between the baPWV and the C-peptide. Second, only one center is included in this study. Our study sample lacks national or regional representation; this limits the ability to generalize the results at an international level. Thus, to improve the consistency of these findings, the study may require replication in other parts of the country or in other regions of the world. Finally, the measurements taken for glucose exposure (fasting, 2 h OGTT, Δ plasma glucose level, and glucose tolerance status) may not capture glucose exposure over time. To that end, future studies addressing this association should be designed to incorporate measures that may better reflect glucose exposure over time.

In conclusion, the results emerging from our study strengthen the available evidence on the importance of the secretion level of islet *ß* cells as a cause and predictor of arterial stiffness in nondiabetic essential hypertensive patients. Furthermore, our data appears highly clinically relevant since knowledge of these pathophysiological mechanisms may allow early interventions on vascular damage, which in turn may prevent clinical events.

## Figures and Tables

**Table 1:** Baseline characteristics of the participants.

Variables	NGT (*n* = 322)	IGT (*n* = 408)	Total (*n* = 730)	*P* value
Age (years)	55.2 ± 13.1	54.5 ± 13.1	54.8 ± 13.1	0.448
Female, *n* (%)	147 (45.7)	182 (44.6)	329 (45.1)	0.778
Current smoking, *n* (%)	74 (23)	111 (27.2)	185 (25.3)	0.396
BMI (kg/m^2^)	26.6 ± 3.9	27.7 ± 4.0	27.2 ± 4.0	<0.001
eGFR (ml min ^−1^ 1.73 m^2^)	104.7 ± 20.5	106.9 ± 22.6	105.9 ± 21.7	0.174
0 h PG (mmol/L)	4.7 ± 0.5	5.1 ± 0.6	4.9 ± 0.6	<0.001
2 h PG (mmol/L)	6.7 ± 0.6	9.3 ± 0.9	8.2 ± 1.5	<0.001
Δ PG (mmol/L)	2.0 ± 0.6	4.2 ± 1.0	3.2 ± 1.4	<0.001
0 h C-peptide (ng/ml)	2.0 (1.7–2.6)	2.3 (1.8–2.9)	2.2 (1.8–2.7)	<0.001
2 h C-peptide (ng/ml)	8.0 (6.3–9.9)	10.2 (8.5–12.7)	9.2 (7.3–11.5)	<0.001
Δ C-peptide (ng/ml)	5.9 (4.5–7.6)	7.7 (6.4–10.2)	7.1 (5.4–8.9)	<0.001
HbA1c (%)	5.9 ± 0.3	6.0 ± 0.4	6.0 ± 0.4	<0.001
TC (mmol/L)	5.1 (4.4–5.8)	5.0 (4.3–5.7)	5.0 (4.3–5.7)	0.420
TG (mmol/L)	1.4 (1.0–2.0)	1.5 (1.1–2.1)	1.5 (1.1–2.1)	0.012
HDL-C (mmol/L)	1.3 ± 0.4	1.2 ± 0.3	1.2 ± 0.3	<0.001
LDL-C (mmol/L)	2.8 (2.4–3.3)	2.8 (2.3–3.3)	2.8 (2.3–3.3)	0.659
Mean 24h heart rate (beats/min)	69.9 ± 8.8	70.9 ± 9.3	70.5 ± 9.1	0.134
Mean 24h SBP (mmHg)	169.8 ± 20.2	172.0 ± 21.4	171.0 ± 20.9	0.156
Mean 24h DBP (mmHg)	107.9 ± 17.0	108.5 ± 16.2	108.2 ± 16.5	0.601
baPWV (cm/s)	1555.3 (1398.8–1749.0)	1556.0 (1371.0–1780.3)	1555.8 (1385.8–1762.6)	0.884
Stable angina, *n* (%)	47 (14.6)	61 (15)	108 (14.8)	0.893
Calcium channel blocker, *n* (%)	226 (70.2)	302 (74.0)	528 (72.3)	0.250
ACEI, *n* (%)	7 (2.2)	13 (3.2)	20 (2.7)	0.405
ARB, *n* (%)	218 (67.7)	271 (66.4)	489 (67)	0.715
*β*-Blocker, *n* (%)	132 (41.0)	170 (41.7)	302 (41.4)	0.855
Diuretic, *n* (%)	69 (21.4)	83 (20.3)	152 (20.8)	0.720
*α*-Blocker, *n* (%)	25 (7.8)	29 (7.1)	54 (7.4)	0.737
Statins use, *n* (%)	199 (61.8)	242 (59.3)	441 (60.4)	0.495

Values are mean ± SD, median (25th and 75th percentiles), or number of patients (percentage of column total). BMI, body mass index; eGFR, estimated glomerular filtration rate; PG, plasma glucose; HbA1c, hemoglobin A1c; TC, total cholesterol; TG, triglycerides; HDL-C, high-density lipoprotein cholesterol; LDL-C, low-density lipoprotein; SBP, systolic blood pressure; DBP, diastolic blood pressure; baPWV, brachial-ankle pulse wave velocity; ACEI, angiotensin-converting enzyme inhibitor; ARB, angiotensin-converting enzyme receptor blocker.

**Table 2:** Univariate correlation with baPWV.

Variables	r	*P* value
Age (years)	0.407	<0.001
Female, *n* (%)	−0.181	<0.001
Current smoking, *n* (%)	−0.151	<0.001
BMI (kg/m^2^)	−0.235	<0.001
eGFR (ml min ^−1^ 1.73 m^2^)	−0.125	0.001
0 h PG (mmol/L)	0.052	0.158
2 h PG (mmol/L)	0.001	0.988
Δ PG (mmol/L)	−0.016	0.675
0 h C-peptide (ng/ml)	−0.043	0.249
2 h C-peptide (ng/ml)	0.086	0.020
Δ C-peptide (ng/ml)	0.115	0.002
HbA1c (%)	0.077	0.037
TC (mmol/L)	0.065	0.079
TG (mmol/L)	−0.100	0.007
HDL-C (mmol/L)	0.158	<0.001
LDL-C (mmol/L)	0.029	0.435
Heart rate (beats/min)	0.025	0.497
Mean 24 h SBP (mmHg)	0.211	<0.001
Mean 24 h DBP (mm Hg)	−0.015	0.678
Stable angina, *n* (%)	0.081	0.029
Calcium channel blocker, *n* (%)	0.160	<0.001
ACEI, *n* (%)	0.009	0.805
ARB, *n* (%)	0.062	0.095
*β*-Blocker, *n* (%)	0.032	0.386
Diuretic, *n* (%)	0.167	<0.001
*α*-Blocker, *n* (%)	0.012	0.751
Statins use, *n* (%)	0.178	<0.001

baPWV, brachial-ankle pulse wave velocity; BMI, body mass index; eGFR, estimated glomerular filtration rate; PG, plasma glucose; HbA1c, hemoglobin A1c; TC, total cholesterol; TG, triglycerides; HDL-C, high-density lipoprotein cholesterol; LDL-C, low-density lipoprotein; SBP, systolic blood pressure; DBP, diastolic blood pressure; ACEI, angiotensin-converting enzyme inhibitor; ARB, angiotensin-converting enzyme receptor blocker.

**Table 3:** Multiple linear regression analysis showing variables independently associated with baPWV.

Variables	Model 1			Model 2			Model 3		
	*β*	95% CI	*P* value	*β*	95% CI	*P* value	*β*	95% CI	*P* value
0 h PG (mmol/L)	0.055	−9.568–−67.659	0.14	0.021	−24.495–−47.317	0.533	0.037	−13.541–−53.059	0.244
2 h PG (mmol/L)	0.032	−8.266–−21.300	0.387	0.039	−5.763–−21.586	0.256	0.035	−5.553–−19.844	0.270
Δ PG (mmol/L)	0.012	−13.292–−18.549	0.746	0.033	−7.477–−22.005	0.334	0.022	−8.710–−18.532	0.479
0 h C-peptide (ng/ml)	−0.062	−44.579–−3.651	0.096	0.037	−10.729–−35.498	0.293	0.091	6.782–−53.487	0.012
2 h C-peptide (ng/ml)	0.077	0.389–−12.906	0.037	0.077	0.865–−12.442	0.024	0.089	2.237–−13.094	0.006
Δ C-peptide (ng/ml)	0.106	3.323–−17.558	0.004	0.078	1.069–−14.318	0.023	0.078	1.534–−13.777	0.014

Model 1: not adjusted. Model 2: adjusted for age and sex. Model 3: adjusted for age, sex, current smoking, BMI, HDL-C, mean 24 h SBP, and TG. CI, confidence interval; baPWV, brachial-ankle pulse wave velocity; PG, plasma glucose; BMI, body mass index; HDL-C, high-density lipoprotein cholesterol; SBP, systolic blood pressure; TG, triglycerides.

## Data Availability

The datasets used and analyzed during the current study are available from the corresponding author on reasonable request.
